# Anxiety and associated factors among prisoners in North West of Amhara Regional State, Ethiopia

**DOI:** 10.1186/s12888-016-0792-y

**Published:** 2016-03-31

**Authors:** Abel Fekadu Dadi, Berihun Assefa Dachew, Teresa Kisi, Nigussie Yigzaw, Telake Azale

**Affiliations:** Department of Epidemiology and Biostatistics, Institute of public Health, University of Gondar, Gondar, Ethiopia; Department of Public Health, College of health sciences, Arsi University, Arsi, Ethiopia; Department of Psychiatry, School of Medicine, University of Gondar, Gondar, Ethiopia; Department of Health Education and Behavioral Science, Institute of public Health, University of Gondar, Gondar, Ethiopia

**Keywords:** Anxiety, Cross-sectional study, Prisoner

## Abstract

**Background:**

Mental illnesses are more common among the prison population than the general public. However, little attention is given to mental health service in low and middle income countries in general. The problem is more so for prisoners where the overall health care is poor. Therefore, the aim of this study was to assess the prevalence of anxiety and the associated factors among prisoners of North West Amhara, Ethiopia.

**Methods:**

Institutional based cross-sectional study was employed from February to March 2015 by taking a sample of 700 prisoners. Simple random sampling method was employed to select three prisons out of 10 prisons found in the North West Amhara region. Generalized Anxiety Disorder 7-item (GAD-7) scale was used to assess prisoners’ anxiety status. The receiver- operator characteristic (ROC) curve was used to determine the cutoff point with high sensitivity and specificity. Structured and pretested interviewer administered questionnaire was used for data collection. Data were checked, coded and entered into Epi Info version 7 and analyzed using R version 3.2.0. Bivariable and multivariable logistic regression analyses were carried out to identify factors associated with anxiety. Odds ratio with its 95 % confidence interval was used as a measure of association. Akaike’s Information’s Criterion (AIC) was used to check model fitness.

**Results:**

A total of 649 prisoners were included in the analysis making the response rate 92.5 %. The prevalence of Anxiety was found to be 36.1 % (95 % CI: 32.7, 39.9). The odds of Anxiety was 2.49(95 % CI: 1.38, 4.55) times higher among prisoners who used to be unhappy in their life before imprisonment. Moreover, smokers were 2.6 (AOR = 2.6, 95 % CI: 1.08, 6.6) times more likely to have anxiety compared with non smokers. However, the odds of the odds of anxiety was 89% lower among Debre-Tabor prisoners (AOR = 0.11, 95 % CI: 0.06, 0.20) and 57 % lower among Gondar prisoners (AOR = 0.43, 95 % CI: 0.28, 0.67).

**Conclusion:**

The prevalence of anxiety is found to be very high among prisoners in North West Ethiopia. Anxiety was associated with current smoking and having had a dissatisfying life. Screening prisoners for common mental disorders and integrated health care is necessary.

## Background

An estimated 10 million people live in prison worldwide to date with the majority of the prison population living in low and middle income countries. The world prison population is growing by about one million per decade [1].

About 450 million people suffer from mental or behavioral disorders globally [2]. These disorders are especially prevalent in prison populations [3]. About one in nine prisoners worldwide suffers from common mental health problems such as depression and anxiety [4].

Epidemiological studies conducted in prisons in several countries have shown a high prevalence of psychiatric morbidity. The prevalence of severe mental disorders can be 5 to 10 times higher than in the general population [5]. In European prisons, the prevalence of psychotic disorders is around 5 %, of depressive or anxiety disorders is around 25 %, and of substance-related disorders is approximately 40 % [6].

Anxiety is a common experience in everyday life. Feeling anxious about certain things is normal and important for adaptation and survival. However, the degree of anxiety that some people feel is sometimes excessive, affects their functional capacity, and can be debilitating. Anxiety disorders are diagnosed when anxiety is either persistent or persistently recurrent, and affects a person’s ability to work, have relationships or interact with others in social situations.

In Ethiopia, approximately 1.7 % of the national health expenditure for 2004 was spent on mental health. The government of Ethiopia has now launched a national mental health strategy that would serve to deliver a comprehensive and integrated service to mental health needs of Ethiopians [7, 8].

Most of the prisoners are in their productive age and are expected to contribute to their country upon their return from the prison Moreover; they can be trained in various vocational activities while in prison that would equip them with knowledge and skills for their future life. This would not happen if the prisoner’s well-being were not maintained.

While these facts remain about mental distress and their contribution to the global burden of diseases, the attention given to mental health is in general and mental health service in the prison in particular is very little in low and middle income countries. There is no accurate count of persons with mental disorder who are incarcerated in Ethiopia and information about prisoners’ health conditions is scarce. However, knowing the mental health needs of prisoners is crucial in order to develop appropriate health care programs for this population [9, 10]. Therefore, this study was aimed to assess anxiety and its associated factors among prisoners in prisons of North West Ethiopia.

## Methods

### Study setting and design

An institution based cross-sectional study was conducted from January to February 2015. Amhara regional state is one of the 11 regions found in the Federal Democratic Republic of Ethiopia. The region covers an area of 20,650,420 square KM and a total population of 19,602,512. While there are 30 prisons in the region, 10 are found in the northwest part. The total number of prisoners in the region was 22,590. Of which 7,564 prisoners were found in the Northwest of the region.

### Sample size determination and sampling procedure

All prisoners found in selected prisons in the North West Amhara regional state were the study populations. Those prisoners who were seriously ill to communicate were excluded from the study. The minimum sample size (n) was computed by single population proportion formula [n = [(Z_a/2_)^2^*P (1-P)]/d^2^] by assuming 95 % confidence level of Z a/_2_ = 1.96, margin of error 5 %, design effect 2, proportion (p) of mental distress among kality prisoners [11] and the final sample size found was 700.

Multi-stage sampling technique was employed to select the study participants. Three prisons: Bahir-Dar, Debre-Tabor, and Gondar were randomly selected from 10 prisons found in the Northwest Amhara Regional state using lottery method. Lists of the prisoners at each of the selected prison were used as a sampling frame. Participants were selected by using computer generated random numbers and proportional to size allocation was used for each prison.

### Data collection tool and data quality control

Data were collected by using structured interviewer administered questionnaire having four parts. These were socio-demographic, socioeconomic, behavioral factors and the Generalized Anxiety Disorder 7-item (GAD-7) scale [12]. The GAD-7 questionnaire is a brief measure of generalized anxiety disorder that assesses problems the respondent bothered by in the past two weeks. The items measure the frequency of symptoms in a scale from 0 (not at all) to 3 (nearly every day). The internal consistency of the tool was checked by conducting reliability test (Cronbach’s Alpha: 0.917).

The questionnaire was translated to the local language, Amharic and back translated to English to check its consistency. Finally it pretested and used after thorough revision was made. Eight trained data collectors with bachelor degree in health were used.

### Data processing and analysis

The collected data were reviewed and checked for completeness and outliers before data entry Data were checked, coded and entered by using Epi Info Version 7 and imported to SPSS version 20 for further cleaning. For measuring the anxiety, ROC curve analysis was done by STATA version 12 in order to determine a cutoff point with high sensitivity and specificity. An individual with a score of 9 or more was considered to have anxiety which was the obtained cutoff value. Bivariate and multivariate logistic regression analyses were carried out using R version 3.2.0. Adjusted odds ratio with its 95 % Confidence interval was used to declare statistical significance between anxiety and associated factors. The variables were entered to the multivariable model using the Backward Stepwise (Likelihood Ratio) regression method. Akaike’s Information’s Criterion (AIC) was used to check model goodness of fit.

## Results

### Socio-demographic characteristics of the prisoners

Six hundred forty-nine prisoners were included in the study with a response rate of 92.7 %. The mean age of the study participants was 30.6 ± 11.49 SD with a mean duration of stay in prison being 9.6 ± 5.5 years. Among the prisoners majority of them were male 583 (89.8 %). Moreover, most of the prisoners, 434 (66.9) came from urban settings. About half, 47.1 % (306) of the prisoners were single (Table 1).

**Table 1:** Socio-demographic characteristics of prisoners among prisoners imprisoned in prisons of Northwest Amhara, 2015

Explanatory variables	Frequency (%)
Sex	
Male	583(89.8)
Female	66(10.2)
Residence	
Urban	434(66.9)
Rural	215(33.1)
Religion	
Orthodox	584(90)
Others(Muslim, catholic & protestant)	65(10)
Marital status	
Single	306(47.1)
Married	228(35.1)
Separated	115(17.7)
Educational status	
Not read and write	108(16.6)
Read and write	97(14.9)
1-8 class complete	129(19.9)
9-12 class complete	206(31.9)
Certificate and above	109(16.8)

### Prison-related characteristics of the study participants

Among the prisoners, 138 (21.3%) were sentenced for life. Three hundred eight (47.5 %) of the prisoners had been practising religious routine. More than half, 389 (59.9 %) of the prisoners were participating in income generating activities. About two-third (62.7 %) of the prisoners were happy with their life before imprisonment The majority (85.8 %) of the prisoners did not believe that the length of time they were penalized was appropriate to their deed. The majority (89.4 %) of the prisoners were moderately satisfied with the care given by correctional institutions (Table 2).

**Table 2:** Shows prisoner related characteristics of the respondents among prisoners imprisoned in prisons of Northwest Amhara, 2015

Explanatory variables	Frequency (%)
Type of prisoner	
Life sentenced prisoner	138(21.3)
Other than life Sentenced prisoner	511(78.7)
Frequency of conduct religious practice	
Always	308(47.5)
Sometimes	229(35.3)
Never	112(17.3)
Participate in income generating activities	
Yes	389(59.9)
No	260(40.1)
Did you have a job before you become prisoner	
Yes	467(72)
No	182(28)
Did you felt happy with your life until you become prisoner	
Yes	567(87.4)
No	82(12.6)
Had you been discriminated because of your imprisonment	
Yes	283(43.6)
No	365(56.4)
How often you feel guilty	
Always	354(54.5)
Sometimes	105(16.2)
Never	190(29.3)
Perceived magnitude of mistake committed	
Hard	304(46.8)
Medium	152(23.4)
Low	193(29.7)
Did you believe on the crime you have committed	
Yes	267(41.1)
No	313(48.2)
I don’t have any idea	69(10.6)
Is the year you penalized is in line with your mistake	
Yes	30(4.6)
No	557(85.8)
I don’t have any idea	62(9.6)
Satisfaction with the care you obtain	
Satisfied	65(10)
Medium satisfaction	580(89.4)
Low satisfaction	4(0.6)

### Mental health related history of the respondents

The majority (85.8 %) of the prisoners did not have a previous history of psychiatric disorder and 87.1 % had no family history of mental illness. Most of the prisoners (81.8 %) were not using any of the substances (Khat, cigarette, or shisha) in the prison (Table 3).

**Table 3:** Shows prisoners related mental health problem of the respondents among prisoners imprisoned in prisons of Northwest Amhara, 2015

Explanatory variables	Frequency (%)
Had previous psychiatric problem	
Yes	92(14.2)
No	557(85.8)
Family history of mental illness	
Yes	84(12.9)
No	565(87.1)
Did you use chat, shisha or cigarette smoking habit	
Yes	118(18.2)
No	531(81.8)
Current cigarette smoking habit	
Yes	31(4.8)
No	618(95.2)
After you released from the prison is there any impossibilities that make you not run the life you had been before	
Yes	213(32.8)
No	436(67.2)
Do you have a hope that you could get excuse	
Yes	414(63.8)
No	235(36.2)
Social support	
Yes	420(64.7)
No	229(35.3)

### Prevalence of anxiety and associated factors

The prevalence of Anxiety among these prisoners was found to be 36.1 % (95 % CI: 23.7, 39.9). After adjusting to socio-demographic, prison related and behavior related factors, the multiple logistic regression model output showed that, leading unhappy life before imprisonment, current cigarette smoking, and place of imprisonment had a significant association with anxiety.

Accordingly, leading unhappy life before imprisonment and anxiety has significant association [AOR = 2.49, 95 % CI, 1.38, 4.55]. Prisoners who were leading unhappy life before imprisonment were 2.49 times more likely to have anxiety. Similarly, smokers were 2.6 [AOR = 2.6, 95 % CI: 1.08 - 6.6] times more likely to have anxiety compared with non smokers. Compared to the prisoners in Bahir-Dar, the odds of having anxiety was less among Debre-Tabor [AOR = 0.11, 95 % CI: 0.06, 0.20] and Gondar prisoners [AOR = 0.43, 95 % CI: 0.28, 0.67) (Table 4).

**Table 4:** Factors associated with anxiety by using multivariable logistic regression among prisoners in Northwest Amhara correctional center

Explanatory variables	Anxiety		COR,_95 % CI_	AOR,_95 % CI_
	Yes (%)	No (%)		
Frequency of conduct religious practice				
Always	103	205	1	
Sometimes	96	133	1.43 (1.00,2.05)	
Never	35	77	0.90 (0.56,1.43)	
Did you feel happy with your life until you became a prisoner				
Yes	193	374	1	
No	41	41	1.94 (1.21,3.09)	2.49 (1.38,4.55)**
Had you been discriminated because of your imprisonment				
Yes	118	165	1	
No	116	250	0.65 (0.47,0.89)	
Did you believe on the crime you made				
Yes	88	179	1	
No	105	208	1.03 (0.72,1.45)	
I don’t know	41	28	2.98 (1.74,5.17)	
Family history of mental illness				
Yes	36	48	1	
No	198	367	0.72 (0.45,1.15)	
Current cigarette smoking habit				
Yes	20	11	3.44 (1.6, 7.6)	2.6 (1.08, 6.6)*
No	214	404	1	1
Is there any impossibilities that prevent you to resettle to the previous state				
Yes	107	106	1	
No	127	309	0.41 (0.29,0.57)	
Do you have a hope that you could get excuse				
Yes	141	273	1	
No	93	142	1.27 (0.91,1.76)	
Name of the prison				
Bahir Dar	136	104	1	1
Debre Tabor	22	168	0.10 (0.06,0.16)	0.11 (0.06,0.20)***
Gondar	76	143	0.41(0.28,0.59)	0.43(0.28,0.67)***

*significant <0.05, **significant *p*< 0.01, ***significant *p*< 0.001

## Discussion

This cross-sectional survey indicated that more than one-third of the prisoners have an anxiety disorder. This result is higher than reported in other low and middle income countries such as India [13] and Chile [14]. However, this is in line with a study conducted among Norwegian [15] and European prisoners [6]. This might be because there is a disparity in Socio-demographic and economic conditions among the study settings. On top of that, there is also a difference in measuring tool with its cutoff value.

The odd of anxiety was 2.49 times higher among prisoners who reported to have had unhappy life before they were imprisoned. This might be because their imprisonment might have added to their life stress that they have had or their anxiety symptoms may have been there for long. The odd of anxiety was 2.6 times higher among current smokers. The association between substance use and mental illnesses has been supported by several studies [14, 15].

The odds of having anxiety were lower by 89 % and 57 % respectively for prisoners of Debre- Tabor and Gondar as compared to those in Bahir-Dar. This may go with the differences in the facilities and the services provided to the prisoners across the institutions. On top of that, Bahir-Dar prison is a regional state prison where prisoners from different parts of the region with more serious cases were to be imprisoned. In line with this, the distresses experienced may be more severe.

Even though the study brought the most interesting topic for discussion, it has got its own limitations. The cross sectional nature of the study limited the chance of showing causal direction between anxiety and the identified factors. The other limitation is related to the tendency of the prisoners to exaggerate symptoms that could overestimate the result.

## Conclusion and recommendation

Anxiety is a common mental health problem among prisoners in the study area. The life circumstances and substance use are among the factors having significant association with anxiety. Screening for common mental disorders and treating prisoners should be given due emphasis.

## Ethics approval and consent to participate

Ethical clearance was obtained from Institutional Review Board of the University of Gondar. Permission to conduct the research was obtained from regional prison administration agency and respective prison offices. Written consent was obtained from the participants after explaining the purpose of the study. To ensure confidentiality, their name and other personal identifiers were not registered in the format. It was explained to the participants that the selection to the study was random and they have the right to not respond for questions that they are not comfortable with. Finally, the questionnaires were kept locked after data entry was completed.

## Availability of data and materials

All relevant data are within the manuscript

## Abbreviations

95 % CI: 95 % confidence interval
AOR: adjusted odds ratio
GAD-7: generalized anxiety disorder 7-item
ROC: receiver- operator characteristic
SPSS: statistical package for social sciences
